# Returning to work after sick leave due to cancer: a 365-day cohort study of Japanese cancer survivors

**DOI:** 10.1007/s11764-015-0478-3

**Published:** 2015-08-30

**Authors:** Motoki Endo, Yasuo Haruyama, Miyako Takahashi, Chihiro Nishiura, Noriko Kojimahara, Naohito Yamaguchi

**Affiliations:** Department of Public Health, Tokyo Women’s Medical University, 8-1 Kawadacho, Shinjuku-ku, Tokyo Japan 162-8666; Department of Public Health, Dokkyo Medical University, Tochigi, Japan; Division of Cancer Survivorship Research, National Cancer Center, Tokyo, Japan; Department of Safety and Health, Tokyo Gas Co. Ltd., Tokyo, Japan

**Keywords:** Cancer survivors, Sick leave, Cumulative return to work (RTW) rate, Partial RTW system, Longitudinal study

## Abstract

**Purpose:**

More employees are experiencing a cancer diagnosis during their working-age years, yet there have been no large-scale Japanese studies investigating sick leave due to cancer. We clarified differences in the cumulative partial and full return to work (RTW) rates between different cancer types among Japanese cancer survivors.

**Methods:**

Data on Japanese employees who experienced an episode of sick leave due to clinically certified cancer diagnosed between 1 January 2000 and 31 December 2011 were obtained from an occupational health register. Subject outcomes within the 365-day period following their initial day of sick leave were utilized for this study. We investigated the cumulative partial/full and full RTW rates by using survival analysis with competing risks and predictors of time to RTW by a Fine-Gray proportional hazard regression model.

**Results:**

One thousand two hundred seventy-eight subjects (1033 males and 245 females) experienced their first episode of sick leave due to cancer during the 12-year follow-up period. Of the subjects, 47.1 % returned to work full time within 6 months of their initial day of sick leave absence, and 62.3 % by 12 months. The cumulative RTW rate varied significantly by cancer type. There were considerable differences in the range of cumulative full RTW rates between the two categories (“lower full RTW rate” groups (“lung,” “hepatic, pancreatic,” “esophageal,” and “blood” cancer groups) vs. “higher full RTW rate” groups (“gastric,” “intestinal,” “breast,” “female genital,” “male genital,” “urinary”): 6.3 to 14.3 % vs. 11.4 to 28.3 % at 60 days, 10.6 to 22.4 % vs. 27.0 to 50.0 % at 120 days, 21.3 to 34.7 % vs. 38.5 to 65.4 % at 180 days, 34.3 to 42.9 % vs. 66.0 to 79.5 % at 365 days). Additionally, older age may be associated with a longer time to full RTW.

**Conclusions:**

More than half of the subjects returned to work full-time within the 365-day period following their initial day of sick leave, with cumulative RTW rates varying by cancer type. Older employees may require a longer time to full RTW.

**Implications of Cancer Survivors:**

It is very important for companies (especially small- and medium-sized companies) to establish and improve their RTW support system for cancer survivors, with knowledge that the median time to RTW is expected to be at least a few months.

## Introduction

Cancer is still one of the leading causes of morbidity and mortality worldwide [1–3]. Public health and medicine for cancer have improved remarkably in recent years [1, 2]. Advances in early diagnosis and effective multidisciplinary treatment have decreased the impact of cancer on the life of cancer survivors [4], and the 5-year survival rate for many kinds of cancers has steadily increased in most developed countries [5–7]. With the aging population, and prolonged cancer survival, the prevalence of cancer survivors is expected to further increase in the near future in most countries [8, 9]. Correspondingly, previous Japanese studies have stated that many cancer patients are surviving longer than in previous decades [10, 11].

The incidence of cancer is higher in senior citizens [1]. However, in fact, according to Japanese cancer surveillance, about 30 % of all diagnosed cancer patients (805,236 patients) belonged to the 20- to 64-year-old working-age group (244,976 patients) in 2010 [12]. As the age of retirement will likely be increased in the future, it is estimated that more working-age employees are expected to experience a cancer diagnosis, as in Western countries [1, 13, 14]. With an increased incidence of cancer diagnoses in the working population, return to work (RTW) of cancer survivors is predicted to be an increasingly relevant situation for the individual, employers, and society [15].

In occupational health studies, RTW rates among cancer survivors vary remarkably in the literature despite comparable study populations [13, 16–18]. From a systematic review of 64 studies, 63.5 % of cancer survivors returned to work after diagnosis [1]. RTW of cancer patients may be viewed as proof of complete recovery, which means that the individuals’ work capacity has recovered to a level that enables a return to work. The situation is generally more complex, affecting occupational rehabilitation and the process of RTW [16, 19]. If employers allow adjustments in work requirements, utilizing occupational health services, partial RTW, etc., RTW may improve the quality of life for cancer survivors by providing social reintegration and increasing self-esteem [20, 21] .

To support effective occupational rehabilitation for cancer survivors, previous studies have focused more on the work adjustments provided after RTW [21]. However, less attention has been focused on the predictors of sickness absence duration due to cancer [9]. To the best of our knowledge, there has been no large-scale study in Japan investigating the work-related outcomes after the initial episode of sickness absence due to cancer, stratified by cancer site [22].

The objective of this study was to clarify differences in the cumulative partial/full and full RTW rates between different cancer sites among Japanese cancer survivors, in a 365-day period following the initial day of sickness absence due to cancer, and to investigate the importance of partial RTW at RTW among Japanese cancer survivors. By specifying the site-stratified cumulative RTW rates, this study may help companies establish and improve their RTW support system for cancer survivors.

## Methods

This was a longitudinal study on the course of sickness absence among employees who were in the process of occupational rehabilitation after a diagnosis of cancer. Employees who experienced an episode of sickness absence due to clinically certified cancer diagnosed between 1 January 2000 and 31 December 2011 were included in this study. During this 12-year period, 1278 employees were diagnosed with cancer, and the 365-day period after their initial day of sickness absence due to cancer was investigated for each subject.

Registered data of sickness absence was obtained from a private occupational health center comprised of approximately 30 occupational physicians (OPs) and 75 occupational health nurses. The occupational health center contracted the OPs with approximately 35 large-scaled Japanese companies of various industries (telecommunications, logistics, energy, construction, etc.), to provide their employees with occupational health services. The total number of employees working for these companies on a full-time basis from 2000 to 2011 was approximately 68,000.

### Sickness insurance system

In Japan, there is no law insuring sickness absence for employees who are not able to work. However, the Labor Contract Act states “A dismissal shall, if it lacks objectively reasonable grounds and is not considered to be appropriate in general societal terms, be treated as an abuse of right and be invalid” [3]. To our knowledge, almost all large Japanese companies have their own sickness insurance system for employees who cannot work due to cancer, depression, stroke, and so on. These sickness insurance systems are only for working individuals, and there is no limitation due to age as long as employees have been working. The time limit for sickness absence varies depending on the company. In the Japanese sickness absence system, part-time sickness absence combined with part-time work is not so common. The fact is that many small- and medium-sized enterprises in Japan do not have such an established sickness insurance system. We guess that cancer survivors who work at small- and medium-sized enterprises have no choice but to quit because of their companies’ economic circumstances, among other factors.

On the other hand, the large companies that we investigated in this study had the same well-established sickness insurance system, associated with their OP contract. The occupational health service registration system of sickness absence and RTW in our study was as follows: after diagnosis of cancer, to certify an episode of sickness absence, an employee was required to submit a physician’s certificate stating that the employee was unable to work due to cancer. In general, the treatment for cancer has a more negative effect on the individual’s work capacity than the cancer itself. The OP confirmed the medical validity of the issued physician’s certificate, and the certificate was sent to the human resources department, of which only the data of full-time workers was registered. The cause of sickness absence was recorded by the OPs, referring to the World Health Organization’s 10th International Classification of Diseases (ICD-10). Under the Labor Standards Law of Japan, employees absent due to sickness were allowed approximately two thirds of their regular salary. In the sickness insurance system, there is no limitation due to age. The time limit for sickness absence due to cancer is set by the OP contract as 3 years. For RTW, employees were required to submit a physician’s certificate stating that they were fit for return to work, as well as participate in interviews with their company’s respective OPs for further confirmation that RTW was medically acceptable. OPs further determined whether the employee in question could return to work full-time (full RTW) or part time (partial RTW, usually 4 to 6 hours a day), and issue the OP’s RTW certificate to the company

As shown in Table 1, the outcome after sickness absence was analyzed by cancer sites, which included more than 50 subjects. Of these, “gastric” cancers were the most prevalent (ICD-10: C16, *n* = 282), followed by “lung” cancers (C33-C34, *n* = 162), and “intestinal” cancers (C17-C21, *n* = 146), which included small intestine cancer (*n* = 7), colon cancer (*n* = 70), and rectal or anal cancer (*n* = 69). The fourth most prevalent was “hepatic, pancreatic” cancers (C22-C25, *n* = 98), which included hepatocellular carcinoma (*n* = 38), cholangiocarcinoma (*n* = 9), gall bladder cancer (*n* = 4), and pancreatic cancer (*n* = 47). The fifth most prevalent was “breast” cancer (C50, *n* = 97), involving only female employees. The sixth most prevalent was “blood” malignancies (C81-C96, *n* = 95), which included leukemia (*n* = 32), malignant lymphoma (*n* = 46), multiple myeloma (*n* = 8), and other related cancers (*n* = 9). The seventh most prevalent was “male genital” cancers (C60-63, *n* = 78), which included prostatic cancer (*n* = 63) and testicular or penis cancer (*n* = 15). Tied for the eighth most prevalent were “esophageal” cancer (C15, *n* = 67) and “female genital” cancers (C51-C58, *n* = 67), which included cancer of the uterus (*n* = 47) and ovarian cancer (*n* = 20). The tenth most prevalent was “urinary” cancers (C64-C68, *n* = 53), which included renal cell carcinoma and ureter carcinoma (*n* = 30), and bladder cancer (*n* = 23). “Other” cancer (*n* = 133) included brain cancer (C71, *n* = 20), oral cancer (C00-C09, *n* = 20), pharyngo-laryngeal cancer (C10-C14, *n* = 27), thyroid cancer (C73, *N* = 19), and others (*n* = 47).

**Table 1:** Basic characteristics of the cancer survivors in this study

Cancer site		Number	Men	Women	Mean age at diagnosis
Gastric		282	262	20	52.9
Esophageal		67	64	3	54.7
Intestinal		146	140	6	51.9
	Small intestine cancer	7	7	0	52.4
	Colon cancer	70	64	6	52.0
	Rectal, anal cancer	69	69	0	51.8
Lung		162	143	19	54.1
Hepatic, pancreatic		98	91	7	54.4
	Hepatocellar carcinoma	38	36	2	52.6
	Cholangiocarcinoma	9	7	2	57.3
	Gall bladder cancer	4	4	0	57.8
	Pancreatic cancer	47	44	3	54.9
Breast		97	0	97	48.1
Female genital		67	0	67	46.4
	Cancer of uterus	47	0	47	47.6
	Ovarian cancer	20	0	20	43.8
Male genital		78	78	0	53.0
	Prostatic cancer	63	63	0	55.7
	Testicular, penis cancer	15	15	0	41.5
Urinary		53	52	1	53.2
	Renal cell carcinoma, ureter carcinoma	30	29	1	52.8
	Bladder cancer	23	23	0	53.6
Blood		95	86	9	49.0
	Leukemia	32	29	3	47.5
	Malignant lymphoma	46	41	5	49.2
	Multiple myeloma	8	8	0	54.0
	Other related cancers	9	8	1	49.0
Other		133	117	16	50.7
	Brain cancer	20	18	2	50.0
	Oral cancer	20	19	1	50.7
	Pharyngo-laryngeal cancer	27	27	0	54.4
	Thyroid cancer	19	9	10	47.9
	Other cancers	47	44	3	49.9
Total		1278	1033	245	51.9

### Statistical analysis

Subject outcomes within the 365-day period following their initial day of sickness absence were obtained from the register and utilized for this study. A 365-day period was arbitrarily chosen for ease of use for other Japanese companies to establish a RTW support system for cancer survivors.

Survival analysis with competing risks was performed to illustrate the cumulative RTW rates by using EZR [23]. We used Fine-Gray proportional hazard regression for competing events in order to analyze whether age, gender, and cancer sites were statistically associated with partial/full RTW and full RTW.

We assigned subjects to 5 categories: “died,” “resigned,” “disabled,” “full RTW,” and “partial RTW.” “Disabled” was defined as subjects who remained absent due to illness by the end of the 365-day period. “Died” and “resigned” were set as factors of competing risks for RTW.

In the Fine-Gray proportional hazard regression, a hazard ratio of more than 1 meant a shorter time to the event, such as full RTW, and a reduced duration of sickness absence until the event, compared with the reference. A hazard ratio of less than 1 meant a longer time to an event. Subjects were stratified by age into four groups by quartiles, resulting in the following age groups: 48 years or younger (reference), 49–52 years, 53–56 years, and 57 years or older.

This study was approved by the Medical Ethics Committee of Tokyo Women’s Medical University (number 3244).

## Results

In the present study, 1278 subjects experienced their first episode of sickness absence due to cancer certified by their physicians, which means that these individuals had not had an episode of sickness absence due to cancer earlier. Shown in Table 1, the characteristics of the subjects were as follows: 1033 (80.8 %) of 1278 subjects were males and 245 (19.2 %) were females. The mean age at the initial day of sickness absence was 51.9 years.

The numbers of those having the event, as well as those who were censored before having the event, are shown in Table 2. After the 365-day period following the initial day of sickness absence, 35 subjects had resigned from their work, 132 subjects had died, and 74 employees had been classified as “disabled,” namely, being unable to return to work within the 365-day period. Approximately 31 of 98 subjects in the “hepatic, pancreatic” cancer group had died within the 365-day period following their initial day of sickness absence. The cancer survivors in the “female genital” cancer group did not die within the 365-day period. Resignation occurred frequently among subjects in the “esophageal cancer” group, with nine subjects resigning from their work within the 365-day period following their initial day of absence. There were no resignations from subjects in the “gastric,” “female genital,” or “urinary” cancer groups. The prevalence of “disabled” subjects was highest in the “blood” malignancies group, in which 19 subjects had a period of sickness absence exceeding 365 days. The “gastric” and “esophageal” cancer groups had the lowest prevalence of “disabled” subjects after the 365-day period. The median time to full RTW among all cancer survivors was 201 days. There were missing values regarding the median time to full RTW among “esophageal,” “lung,” “hepatic, pancreatic,” and “blood” malignancy groups because the cumulative full RTW rates did not reach 50 %. Approximately 3.5 times more subjects returned to work partially compared to full-time. When stratified by cancer site, the partial to full RTW ratio ranged from 1.6 (others) to 8.4 (esophageal cancer). As shown in Table 2, the median duration of sickness absence until either partial or full RTW was 80 days. The median duration until full RTW was 201 days (about 6.5 months).

**Table 2:** Occupational register outcomes within 365 days after initial day of sickness absence, stratified by cancer site

Cancer site	Number	Median time to partial/full RTW (days)	Median time to full RTW (days)	1. Died, *N*	2. Resigned, *N*	3. Disabled, *N*	4. RTW, *N*	Full RTW, *N*	Partial RTW, *N*	Partial RTW/full RTW (ratio)
Gastric	282	62	124	16	0	3	263	40	223	5.6
Esophageal	67	123	–	7	9	2	47	5	42	8.4
Intestinal	146	66.5	136.5	16	3	4	123	31	92	3.0
Lung	162	96.5	–	22	7	11	122	31	91	2.9
Hepatic, pancreatic	98	194	–	31	6	7	54	13	41	3.2
Breast	97	91	209	1	2	6	87	15	72	4.8
Female genital	67	83	172	0	0	5	62	11	51	4.6
Male genital	78	60.5	24.5	1	4	5	68	16	52	3.3
Urinary	53	52	127	7	0	1	45	15	30	2.0
Blood	95	241	–	12	1	19	62	14	48	3.4
Other	133	91	195	19	3	11	98	38	60	1.6
Total	1278	80	201	132	35	74	1031	229	802	3.5

**Fig. 1:**
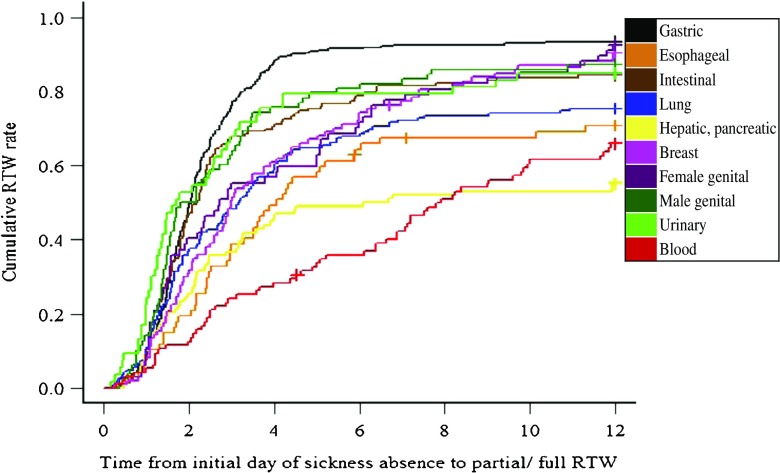
Survival analysis with competing risks for time to partial or full RTW (months). The *crosses* represent censoring due to loss to follow-up

Figure 1 shows the duration of time until partial or full RTW for each cancer site. As the result of survival analysis with competing risks, the overall cumulative RTW rates after the initial day of sickness absence at 60, 120, 180, and 365 days were 41.0, 64.1, 71.6, and 80.9 % respectively.

**Fig. 2:**
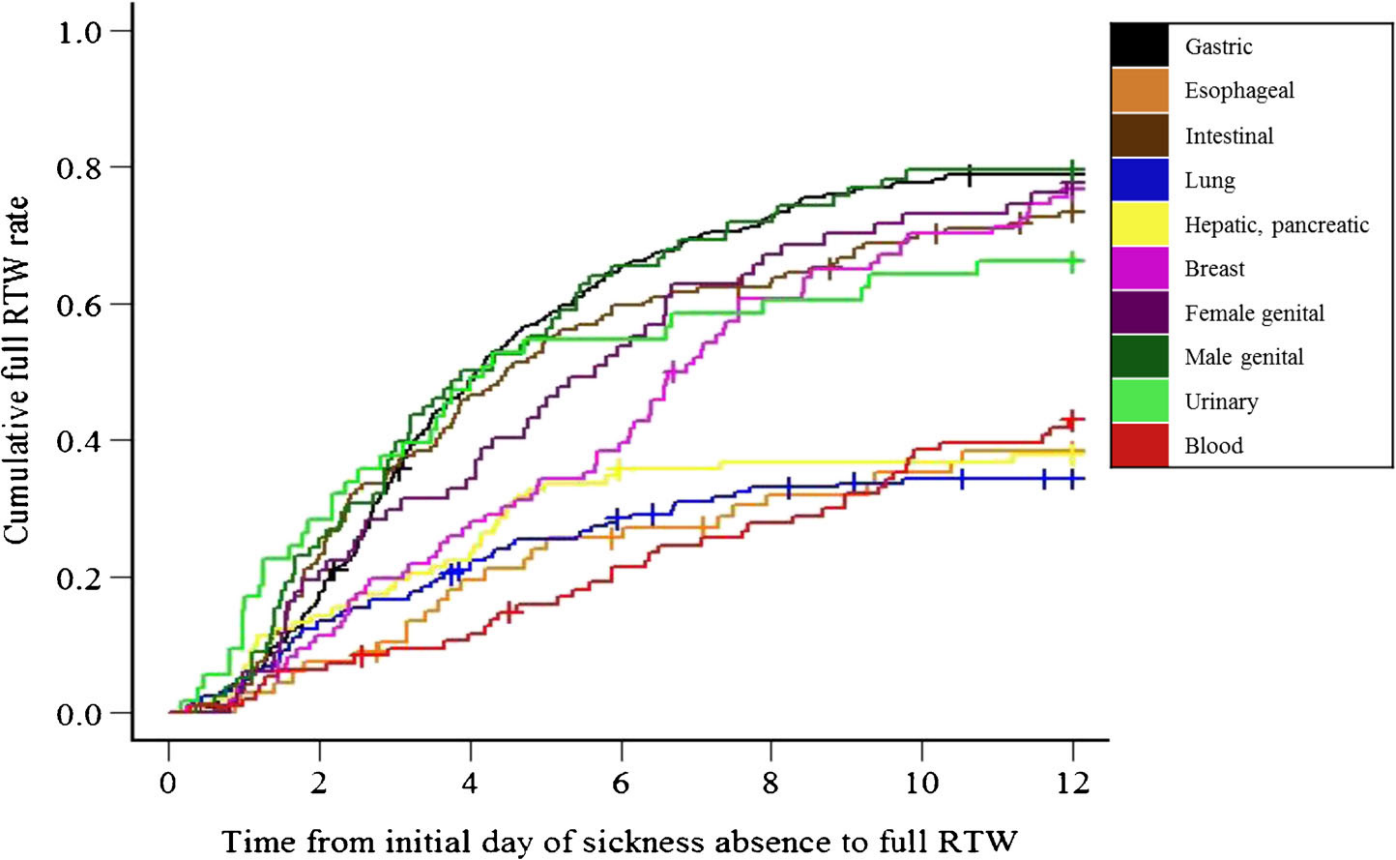
Survival analysis with competing risks for time to full RTW (months). The *crosses* represent censoring due to loss to follow-up

Figure 2 shows the duration of time until full RTW. As the result of survival analysis accounting for competing risks, the cumulative full RTW rates at 60, 120, 180, and 365 days were 16.7, 34.9, 47.1, and 62.3 %, respectively. The ten different cancer site groups were approximately divided into two categories: those with a lower cumulative full RTW rate, consisting of the “lung,” “hepatic, pancreatic,” “esophageal,” and “blood” cancer groups, and those with a higher rate (“gastric,” “intestinal,” “breast,” “female genital,” “male genital,” “urinary”). There were considerable differences in the range of cumulative full RTW rates between the two categories (“lower full RTW rate” groups vs. “higher full RTW rate” groups: 6.3 to 14.3 % vs. 11.4 to 28.3 % at 60 days, 10.6 to 22.4 % vs. 27.0 to 50.0 % at 120 days, 21.3 to 34.7 % vs. 38.5 to 65.4 % at 180 days, 34.3 to 42.9 % vs. 66.0 to 79.5 % at 365 days). The group with the lowest cumulative RTW rate (both total RTW and full RTW rates) was the “blood” malignancy group, whose cumulative total RTW rates at 60, 120, 180, and 365 days were 12.6, 27.4, 35.9, and 65.8 %, and whose cumulative full RTW rates were 6.3, 10.6, 21.3, and 42.9 %, respectively.

As shown in Table 3, as for cancer sites, employees with “esophageal,” “lung,” “hepatic, pancreatic,” and “blood” cancers had a longer time to partial/full RTW than those with “gastric” cancer.

**Table 3:** Fine-Gray regression model for the time to partial/full RTW within 365 days

		Univariable analyses		Multivariable analyses	
Variables	Categories	HR (95 % CI)	*P* value	HR (95 % CI)	*P* value
Age (years)					
	<48 (ref)	1		1	
	49–52	0.96 (0.79–1.18)	0.68	0.96 (0.78–1.18)	0.68
	53–56	0.99 (0.85–1.15)	0.92	1.02 (0.87–1.20)	0.77
	>57	0.86 (0.73–1.02)	0.09	0.90 (0.75–1.07)	0.24
Sex					
	Men (ref)	1		1	
	Women	0.90 (0.78–1.03)	0.11	0.85 (0.67–1.09)	0.21
Cancer sites	Gastric (ref)	1		1	
	Esophageal	0.54 (0.40–0.72)	<0.01	0.53 (0.40–0.72)	<0.01
	Intestinal	0.94 (0.75–1.16)	0.55	0.93 (0.75–1.16)	0.53
	Lung	0.68 (0.55–0.84)	<0.01	0.68 (0.55–0.84)	<0.01
	Hepatic, pancreatic	0.40 (0.29–0.54)	<0.01	0.40 (0.30–0.55)	<0.01
	Breast	0.82 (0.67–1.00)	0.06	0.70 (0.52–0.95)	0.02
	Female genital	0.89 (0.70–1.13)	0.34	0.76 (0.55–1.06)	0.10
	Male genital	1.03 (0.78–1.36)	0.83	1.04 (0.79–1.38)	0.77
	Urinary	1.05 (0.73–1.52)	0.78	1.06 (0.74–1.53)	0.75
	Blood	0.41 (0.32–0.52)	<0.01	0.40 (0.32–0.51)	<0.01

As shown in Table 4, Fine-Gray regression analysis revealed that female subjects were not statistically associated with a longer time to full RTW (hazard ratio of female for time to full RTW was 1.07, 95 % confidence interval 0.78–1.46), compared to male subjects. According to the multivariate analysis including all variables, subjects in the >57 year age groups had longer times to full RTW than the <48 year (reference) age group (the hazard ratio for RTW was 0.78, 95 % confidence interval 0.64–0.97). In addition, those with “esophageal,” “lung,” “hepatic, pancreatic,” and “blood” cancers had a longer time to full RTW than those with “gastric” cancer.

**Table 4:** Fine-Gray regression model for the time to full RTW within 365 days

		Univariable analyses		Multivariable analyses	
Variables	Categories	HR (95 % CI)	*P* value	HR (95 % CI)	*P* value
Age (years)					
	<48 (ref)	1		1	
	49–52	0.84 (0.67–1.05)	0.13	0.89 (0.70–1.12)	0.31
	53–56	0.80 (0.67–0.95)	0.01	0.87 (0.73–1.04)	0.14
	>57	0.71 (0.58–0.86)	<0.01	0.78 (0.64–0.97)	0.02
Sex					
	Men (ref)	1		1	
	Women	0.88 (0.75–1.03)	0.12	1.07 (0.78–1.46)	0.69
Cancer sites	Gastric (ref)	1		1	
	Esophageal	0.35 (0.24–0.53)	<0.01	0.36 (0.24–0.54)	<0.01
	Intestinal	0.99 (0.79–1.24)	0.18	0.98 (0.78–1.23)	0.86
	Lung	0.33 (0.24–0.45)	<0.01	0.29 (0.21–0.39)	<0.01
	Hepatic, pancreatic	0.38 (0.26–0.55)	<0.01	0.39 (0.27–0.56)	<0.01
	Breast	0.86 (0.69–1.08)	0.19	0.86 (0.60–1.24)	0.43
	Female genital	0.99 (0.76–1.30)	0.96	0.99 (0.67–1.46)	0.95
	Male genital	1.18 (0.90–1.55)	0.24	1.18 (0.89–1.55)	0.25
	Urinary	0.90 (0.61–1.34)	0.61	0.90 (0.61–1.33)	0.61
	Blood	0.38 (0.28–0.52)	<0.01	0.37 (0.27–0.51)	<0.01

## Discussion

The growing prevalence of employees diagnosed with cancer has increased the necessity for better RTW support systems for cancer survivors, particularly for occupational health. To the best of our knowledge, this was the first large-scale study in Japan identifying factors that influence cumulative RTW rates among cancer survivors, using survival analysis.

We found that 71.6 % of subjects returned to work (part-time or full-time) within 6 months of their initial day of sickness absence, and 80.9 % by 12 months. This is in accordance with findings from other Western studies stating that many cancer survivors return to work after sickness absence, and working ability improves over time [24–27]. For full RTW, 47.1 % of subjects returned to work full-time within 6 months of their initial day of sickness absence, and 62.3 % by 12 months in the present study, while a previous study reported rates of 60–89 % [27]. This discrepancy may be caused by differences in study population, design, and methodology [17, 28, 29].

Furthermore, as the result of our study considering competing risks, only 4.1 % of subjects resigned from their place of employment within 1 year of their initial day of sickness absence due to cancer, while approximately 8 % of cancer survivors resigned within 2 years in the Netherland’s study [17].

### Cumulative RTW rate by cancer site

The cumulative RTW rate varied significantly by cancer site, in line with previous reports [17, 24, 28, 30]. The previous studies reported that lung cancer and blood malignancies had lower total RTW rate than other cancer sites [17, 31]. The differences observed in RTW rates between previous studies may be explained by differences in the company health care systems, and the particular country’s focus on preventive care [32].

Our study showed that the “blood” malignancy group had a lower cumulative RTW rate. Other studies have previously stated that blood malignancies were the most difficult cancers for cancer survivors to achieve RTW [19, 33]. In the aspect of clinical oncology, chemotherapy is indicated for all patients of blood malignancy, according to the Japanese Clinical Guidelines of Hematopoietic Tumor [34]. Chemotherapy for blood malignancies such as acute myeloid leukemia and Hodgkin’s disease is quite effective, whereas effectiveness may be significantly lower for other cancer types [35]. However, it has been reported that patients with blood malignancies may be more severely affected by the side effects of chemotherapy [35]. One study reported that chemotherapy, which normally continues for several months, decreases the quality of life of cancer patients, leading to symptoms such as general malaise, distress, mental disorders [36, 37]. Prevention of depression may be one of the most important factors impacting RTW. Depression has been reported to have negative and long-time effects on cancer survivors, decreasing the general quality of life, worsening compliance with chemotherapy, and lengthening the duration of hospitalization [38, 39]. While a decrease in cumulative RTW rate may be due to the inclusion of patients with worse prognoses, chemotherapy itself may be one noteworthy factor influencing the duration of sickness absence [17, 25, 26, 40].

### Time from initial day of sickness absence to RTW

The present study showed that cancer site was strongly associated with time to RTW, consistent with previous studies [41, 42], with the cumulative RTW rates of the “esophageal,” “lung,” “hepatic, pancreatic,” and “blood” cancer groups lower than the other groups.

The median time to partial or full RTW for each cancer site group, other than the “esophageal” and “blood” cancer groups, was approximately 3 months. By using a patient survey conducted in 2008 by the Ministry of Health, Labor and Welfare, the mean duration of hospitalization was compared with the median time to partial or full RTW for patients of each cancer site group; gastric cancer 27 days (duration of hospitalization) vs 62 days (duration of sickness absence), esophageal cancer 24 vs 123 days, lung cancer 26 vs 96.5 days, and breast cancer 16 vs 91 days [43]. These differences may be due to outpatient treatment (chemotherapy, radiotherapy, etc.), psychiatric impairment, or other factors.

The rate of RTW declined over time after the initial day of sickness absence; the RTW rate was highest in the first quarter of the year, followed by the second quarter, a tendency in accordance with previous studies [9, 17, 28, 31]. This may be due to the shape of the distribution of sickness absence, which has been reported to be heavily right-skewed [29]. While Christensen et al. reported that RTW rates decrease with increasing duration of sickness absence, occupational rehabilitation at the early stages of sickness absence may remain important [44].

In the present study, the Fine-Gray regression model for the total population demonstrated subjects in the >57 year age groups had a longer duration of sickness absence until full RTW compared to the <48 year age group. Some previous studies have reported that older cancer survivors require a longer time to RTW, whereas other studies were unable to show significant correlation between age and time to RTW [19, 41]. Roelen et al. reported age was correlated with full RTW in genital cancer survivors, but not for other cancers [17].

The present study showed that there was no significant association between time to full RTW and gender, in contrast with previous studies stating that “female” was negatively associated with RTW [1, 15, 41]. In a French study on the RTW process within 2 years of cancer diagnosis, the authors hypothesized that the difference observed between men and women may have been due to an increased incentive for men to RTW because of economic responsibility for their families [9]. A Dutch study demonstrated that gender was only associated with time to full RTW in patients diagnosed with a blood malignancy, observing that women took a longer time to full RTW compared with men [17].

### Partial RTW and full RTW

In addition to the previous discussion, there were other large differences between the present Japanese study and previous Western ones [24, 28, 45]. The present study observed that many subjects returned to work part-time; in contrast, many of the subjects in the previously published studies returned to work full-time [24, 31, 39, 41]. Comparison of the present results with previous studies may be difficult using the values including both partial and full RTW.

The difference in cumulative RTW rates between Figs. 1 and 2 demonstrate that a “partial RTW system” for cancer survivors seemed to markedly improve cumulative RTW rates, except for the “blood” malignancies group. Thus, establishment of a partial RTW system in companies (especially small- and medium-sized companies) may improve cumulative RTW rates.

The ratio of partial RTW to full RTW among cancer site groups was highest in the “esophageal” cancer group, which may have possibly been due to a restriction in diet following esophageal surgery, leading to less physical strength and prevention of full-time work. However, decision of partial or full RTW was not based on an objective standard; rather, it was entirely based on the OPs’ subjective judgment. In general, RTW for cancer survivors is quite complex, depending on a variety of medical and non-medical factors [41]. Previous reports have stated that factors influencing RTW include socio-demographic factors (age, gender), disease-specific variables (site, stage, treatment, comorbidity), and social-environmental elements [45, 46]. As for disease-specific variables, various levels of symptom severity are associated with varying patterns of work disability [16]. In the present study, OPs were assumed to understand the disease-specific information of each subject for their RTW, and the decision of partial RTW may be associated with findings of a worsened prognosis, such as existence of metastasis [47].

Furthermore, we used Fine-Gray regression analysis in order to investigate predictors of the time to full RTW, but only studying full RTW underestimates the proportion of individuals who return to the labor market. These results show that this is especially so for cancer sites with a high partial/full RTW ratio (e.g., gastric and esophageal cancers).

### Strengths and limitations

One of the strengths of the present study was the enrollment of a large group of subjects; approximately 1300 Japanese employees who experienced a period of sickness absence due to cancer were included in the study, the first large-scale Japanese RTW study of cancer survivors. Additionally, the follow-up rate was very high (almost 100 %) because the sickness-absence register was company-enforced, with OPs consistently recording all cancer cases arising in the workforce and certifying sickness absences. With this system, there was less subject selection and loss to follow-up biases that may have possibly affected other studies. Furthermore, we used an objective measurement of sickness absence; the present study was based on data from clinically certified sickness absence using physicians’ certificates. Utilization of clinically made ICD-10 diagnoses of the subjects’ cancers allowed for a higher validity and reliability than categorization by other diseases, such as psychiatric diseases. Another strength of our study was that we used the partial RTW system, investigating “the partial/full RTW ratio judged by OPs at RTW” and “the cumulative partial/full RTW rate.”

When interpreting the results of the present study, several limitations should be noted. First, the medical information of the subjects was not available for use in the present study, such as stage of cancer, pathological degree of malignancy, and type of treatment (surgery, chemotherapy, radiation therapy). Second, we could not deny the existence of comorbidities in the subjects, due to the registration of only one diagnosis per episode of sickness absence by the OPs. Subjects may have had other disorders during the sickness absence, such as depression or ileus after iliac surgery, or other symptoms such as depressive mood, anxiety, or sleep disorders, often found in cancer survivors. Knowledge of comorbidities is necessary due to their influence on time to RTW [24]. Third, no differentiation was made between subjects who may have had previous episodes of cancer prior to working at the company in question, or subjects who experienced recurrence or other types of cancer after the study period. Fourth, because the majority of the subjects were male, caution is necessitated for generalizations across the entire workforce based on the present results. Fifth, it is very important to note that the initial date of sickness absence may have been significantly different from the date of diagnosis, or the date of the start of the illness. Sixth, it might be suspicious to have the proportional hazard assumption according to the visual inspection of the output. However, the logistic regression model showed, the same as the Fine-Gray proportional hazard regression model, that “57 years or older (reference: 48 years or younger),” “esophageal cancer (reference: gastric cancer),” “lung cancer,” “hepatic, pancreatic cancer,” and “blood malignancies” had lower probabilities of full RTW. Seventh, different forms of cohort biases might be introduced. This is because all individuals are pooled in the analyses, regardless of their year of sickness absence. Since there have obviously been changes in cancer treatments, RTW policies, and other factors over the years, this could (if there are systematic differences) be a problem.

### Future studies

Further clarification of the predictors of sickness absence due to cancer is required to better support the drafting of a RTW strategy for cancer survivors. The following predictors of time to RTW require further investigation: disorder-related factors (diagnosis, stage of cancer, content of medical treatment) and environmental factors (job demand, supervisory support, and co-worker support). Recurrent sickness absence after RTW among cancer survivors should also be investigated for tertiary prevention, in particular, recurrent sickness absence due to psychiatric disorders (such as depression, anxiety disorders) after RTW. Research on mental health problems may be important for the improvement of quality of life for cancer survivors.

## Conclusion

More than half of the total cancer survivors returned to work within the 365-day period following their initial day of sickness absence. The cumulative RTW rate was dependent on the type of cancer. Older employees may require a longer time to full RTW. Occupational health professionals may better support cancer survivors for RTW, with the knowledge that cumulative RTW rates vary by cancer type. For cancer survivors, it is very important for companies (especially small- and medium-sized ones) to establish and improve their RTW support systems (e.g., partial RTW system), with the knowledge that the median time to RTW is expected to be at least a few months.
